# MG149 inhibits histone acetyltransferase KAT8-mediated IL-33 acetylation to alleviate allergic airway inflammation and airway hyperresponsiveness

**DOI:** 10.1038/s41392-021-00667-4

**Published:** 2021-09-08

**Authors:** Yahui Liu, Juan Du, Xinnan Liu, Lingbiao Wang, Yichao Han, Chunrong Huang, Rui Liang, Fang Zheng, Guochao Shi, Bin Li

**Affiliations:** 1 Department of Respiratory and Critical Care Medicine, Ruijin Hospital, Shanghai Jiao Tong University School of Medicine, Shanghai, China; 2 Department of Immunology and Microbiology, Shanghai Institute of Immunology, Shanghai Jiao Tong University School of Medicine, Shanghai, China; 3 Division of Rheumatology, Huashan Hospital, Fudan University, Shanghai, China; 4 Department of Thoracic Surgery, Ruijin Hospital, Shanghai Jiao Tong University School of Medicine, Shanghai, China; 5 Department of Immunology, School of Basic Medicine, Tongji Medical College, Huazhong University of Science and Technology, Wuhan, China

**Keywords:** Inflammation, Epigenetics

Asthma is one of the most common heterogeneous airway diseases. Worldwide, the prevalence of doctor-diagnosed asthma in adults was reported to be 4.3% in 2003.^1^ In China, the overall prevalence of asthma during 2012 and 2015 was 4.2%, representing 45.7 million Chinese adults.^2^ Although comprehensive approaches have been used in clinical practice, a considerable number of people are still hospitalized for acute exacerbation of asthma every year. In China, 15.5% of asthmatics reported at least one emergency room visit and 7.2% reported at least one hospital admission due to exacerbation of respiratory symptoms.^2^ A need exists to clarify the mechanism of asthma’s pathogenesis and to identify more appropriate and precise therapies for asthma.

Post-translational modifications (PTMs) are involved in many physiological and pathological processes by regulating the stability, localization, and activity of proteins. The lysine acetylation modification has emerged as one of the major PTMs, which regulates gene transcription and various cellular functions. Previous studies have found an increase in lysine acetyltransferase (KAT) activity and some reduction in lysine deacetylase (KDAC) activity in asthma, which implies that these enzymes may be potential drug targets in asthma.

Histone acetyltransferase KAT8 is a member of the MYST family of proteins, which is highly conserved across species and involved in a wide range of cellular functions, including gene expression, DNA damage repair, cell death, stem cell development, and oncogenesis. However, little is known about whether KAT8 plays a role in asthma and how it works. There is only one report on this topic, in which Bosch et al. found that pro-inflammatory gene expression was decreased in lipopolysaccharide and interferon-gamma stimulated murine precision-cut lung slices after MG149 (a KAT8 inhibitor) treatment, which indicated that MG149 had the potential to develop applications for the treatment of inflammatory lung diseases.^3,4^ Whether MG149 can play role in relieving inflammation in vivo and how it exerts an anti-inflammatory effect remain unclear.

In order to explore the effects of MG149 on airway inflammation, we developed a house dust mite (HDM)-challenged mouse model of allergic asthma (Supplementary Fig. S1a). For the MG149 intervention group, the anaesthetized C57BL/6 mice were pretreated with MG149 60 minutes prior to HDM administration. We found that mice exposed to HDM developed airway hyperresponsiveness (AHR), and administration of MG149 significantly reduced the airway resistance (Supplementary Fig. S1b). Additionally, administration of MG149 also significantly decreased total protein levels and total cell numbers in bronchoalveolar lavage fluid (BALF) compared with HDM-challenged mice (Supplementary Fig. S1d, e). Hematoxylin and eosin (HE) and periodic acid Schiff (PAS) staining showed that MG149 could relieve the inflammatory cell infiltration and mucus secretion (Supplementary Fig. S1f, g). Additionally, we also found that MG149 treatment significantly reduced collagen deposition around the airways (Supplementary Fig. S1h). Collectively, these results demonstrated that MG149 could relieve HDM-induced AHR and airway inflammation in a murine allergic asthma model.

Next, we sought to study the mechanism underlying KAT8’s function in this process. Western blotting of mouse lung homogenate demonstrated that HDM treatment increased the expression of IL-33, an effect that was reduced by administration of MG149 (Supplementary Fig. S2a). The changes of IL-33 protein levels were consistent with the results of immunohistochemistry (IHC) of lung sections and enzyme-linked immunosorbent assay (ELISA) of mouse lung homogenate (Supplementary Fig. S2b–d). Given that IL-33 was a key cytokine participating in the pathogenesis of asthma, we hypothesized that IL-33 was related to this process by which KAT8 inhibition alleviated allergic asthma. To verify this hypothesis, we developed allergic asthma model in both wild-type (WT) and *Il33*^*−/−*^ mice. The observed series of changes in airway resistance, total IgE, total BALF cell numbers, and total BALF protein levels in WT mice after HDM stimulation and MG149 treatment were the same as before, but it was intriguing that MG149 did not play a role in the absence of IL-33 (Fig. 1a, b, Supplementary Fig. S3). Next, we measured the type 2 cytokines in mediastinal lymph nodes (mLNs) and lung tissue to further assess type 2 immune response and the efficacy of MG149. Similar results were found in that there was no difference between HDM-treated *Il33*^−^^*/−*^ mice and HDM + MG149 treated *Il33*^−^^*/−*^ mice (Fig. 1b–f, Supplementary Figs. S4–8). Taken together, these data indicated that KAT8 played a role in asthma, and this process was dependent on IL-33.

**Fig. 1:**
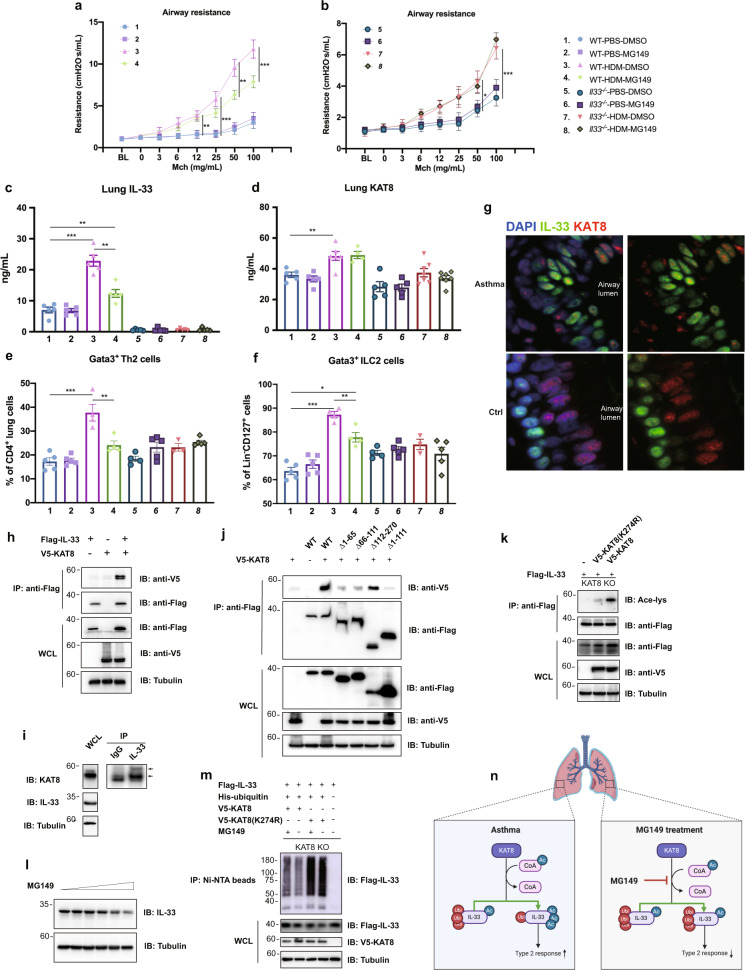
MG149 inhibits histone acetyltransferase KAT8-mediated IL-33 acetylation to alleviate allergic airway inflammation and airway hyperresponsiveness Airway responsiveness to methacholine (Mch) in wild-type (WT) mice (**a**) and *Il33* knockout mice (**b**) exposed to intranasal PBS or HDM and intraperitoneal DMSO or MG149 was measured (*n* = 5–7 mice per genotype and treatment). Protein levels of IL-33 (**c**) and KAT8 (**d**) in lung homogenate of WT and *Il33* knockout mice were measured by enzyme-linked immunosorbent assay (ELISA) (*n* = 4–7). The proportion of Gata3^+^ Th2 cells (**e**) and Gata3^+^ ILC2 cells (**f**) in lung tissue of WT and *Il-33* knockout mice were measured via flow cytometry (*n* = 3–5). **g** Representative confocal images of lung tissues from asthma patients and controls (surgical specimens of lobectomy or segmentectomy patients) labeled for IL-33 (green), and KAT8 (red). The nuclei were stained with DAPI (blue). *600×*. **h** Interaction of IL-33 and KAT8. Exogenous co-immunoprecipitation (co-IP) was performed with the anti-Flag antibodies in HEK293T cells, followed by immunoblot. **i** Endogenous co-IP was performed with anti-IL-33 antibody or pre-immune control serum (IgG) in BEAS-2B cells, followed by immunoblot. **j** Co-IP analysis for the interaction between full-length KAT8 and different truncations of IL-33 via immunoprecipitation with anti-Flag, followed by immunoblot. **k** Acetylation levels of exogenous IL-33 were detected via co-transfection of Flag-IL-33 with wild type V5-KAT8 or V5-KAT8 mutant K274R in KAT8 knockout HEK293T cells. **l** Inhibition of endogenous KAT8 via adding MG149 (1-10 μM) reduced endogenous IL-33 levels in BEAS-2B cells. **m** His pull-down assay in KAT8 knockout HEK293T cells showed that WT KAT8 reduced ubiquitination of IL-33, and MG149 could partially restore ubiquitination of IL-33. **n** Schematic representation of anti-inflammatory mechanism of MG149 in allergic asthma. Error bars represent standard error of the mean (SEM). Data are mean ± SEM. Each symbol (**c**–**f**) represents an individual mouse. Comparisons were made using unpaired t test. Data were from three independent experiments. *P < 0.05, **P < 0.01, ***P < 0.001

We next explored how KAT8 functioned in asthma. We performed confocal microscopy to localize KAT8, and found that the airway epithelial cells of asthmatics highly expressed IL-33, while the control group only expressed IL-33 in the basal cells of the airways (Fig. 1g, Supplementary Fig. S9). More importantly, KAT8 was also expressed highly in the lungs of asthma patients and co-localized with IL-33 (Fig. 1g, Supplementary Fig. S9). We also localized KAT8 in mouse lung sections and found that IL-33 and KAT8 were also co-localized in mouse alveolar epithelial cells (Supplementary Fig. S10). These results suggested that KAT8 was spatially co-localized with IL-33, which further confirmed the possible interaction between the two proteins.

In view of the above, we suspected that KAT8 may interact with IL-33. First, we constructed overexpression vectors of mouse IL-33 and KAT8, and found that mouse IL-33 interacted with KAT8 via co-immunoprecipitation (coIP) (Supplementary Fig. S11a). Because IL-33 and KAT8 were both highly conserved proteins, we speculated that human IL-33 and KAT8 may also interact. We then performed coIP to confirm the interaction between human IL-33 and KAT8 in human embryonic kidney (HEK) 293 T cells (Fig. 1h, Supplementary Fig. S10b), and we verified this interaction using antibodies against endogenous IL-33 in BEAS-2B cells (Fig. 1i). To further map the IL-33 domain required for the interaction with KAT8, we created different truncated forms of IL-33 (Supplementary Fig. S11c). Among these truncations, the one lacking the IL-1-like cytokine domain had the strongest combination with KAT8 (Fig. 1j, Supplementary Fig. S11d, e). Taken together, these findings suggested a specific interaction between IL-33 and KAT8.

Since IL-33 could interact with KAT8 and KAT8 is a type of histone acetyltransferase, we then analyzed whether IL-33 was an acetylation substrate of KAT8. It has already been demonstrated that K274R or K274A mutants could decrease the histone acetyltransferase activity of KAT8.^5^ Next, we detected IL-33 acetylation in KAT8 knockout HEK293T cells transfected with WT KAT8 and KAT8 K274R mutant. WT KAT8 could increase IL-33 acetylation, while KAT8 K274R mutant had a weak effect (Fig. 1k). Collectively, these data implied that KAT8 interacted with IL-33, and IL-33 is acetylated by KAT8.

Next, we explored the effect of acetylation on IL-33. We found that the N-terminal domain of IL-33 was responsible for IL-33’s interaction with KAT8, and was mainly related to the stability of IL-33. Therefore, we investigated the effects of acetylation on the protein stability of IL-33. We found that the acetylation level of exogenous IL-33 was increased in Trichostatin A (TSA)-treated ﻿HEK293T cells (Supplementary Fig. S12a). Also, using antibodies against Flag, we verified that TSA could stimulate acetylation of exogenous IL-33 (Supplementary Fig. S12b). Both TSA and Nicotinamide (NAM) treatment could increase the expression of exogenous IL-33, and the effects of TSA were more obvious (Supplementary Fig. S12c). TSA could also induce acetylation of endogenous IL-33 in Normal and Diseased Bronchial Epithelial (NHBE) cells and BEAS-2B cells and increase the expression of endogenous IL-33 (Supplementary Fig. S12d–f). Next, we studied the relationship between KAT8 and the protein stability of IL-33. We found that exogenous KAT8 could increase the expression of exogenous IL-33, and MG149 treatment could reduce the expression of endogenous IL-33 (Fig. 1l, Supplementary Fig. S12g, h). Our previous studies showed that IL-33 could also be ubiquitinated. Knowing that ubiquitination is a key mechanism to regulate the proteasome-mediated proteolysis of target proteins, we theorized that IL-33 acetylation might affect IL-33’s stability by decreasing the ubiquitination of IL-33. To this aim, expression vectors encoding Flag-IL-33, His-ubiquitin, and V5-KAT8/V5-KAT8(K274R) were transfected into KAT8 knockout HEK293T cells. We found that KAT8 could reduce the polyubiquitination of IL-33, and this reduction could be retrieved by adding MG149 (Fig. 1m). Taken together, these results suggested that KAT8 enhanced IL-33’s stability by reducing its polyubiquitination.

In summary, we found that IL-33 is a substrate of KAT8. KAT8 can acetylate IL-33 and enhance protein stability via reducing its polyubiquitination. Inhibition of KAT8 can alleviate allergic airway inflammation and AHR in an asthma model, an effect that was mediated by decreasing the levels of IL-33 in mice (Fig. 1n). These findings can provide a new alternative strategy for asthma treatment.

## Supplementary information

Supplementary Materials for MG149 inhibits histone acetyltransferase KAT8-mediated IL-33 acetylation to alleviate allergic airway inflammation and airway hyperresponsiveness

## Data Availability

All data relevant to this work are included in this paper and Supplementary Information.
