# Supplementary material for: MG149 inhibits histone acetyltransferase KAT8-mediated IL-33 acetylation to alleviate allergic airway inflammation and airway hyperresponsiveness
Source: Signal Transduct Target Ther. 2021 Sep 8;6:321. doi: 10.1038/s41392-021-00667-4 (PMC8423820; doi:10.1038/s41392-021-00667-4)
Supplement: Supplementary file 1 — Supplementary Materials for MG149 inhibits histone acetyltransferase KAT8-mediated IL-33 acetylation to alleviate allergic airway inflammation and airway hyperresponsiveness [file 41392_2021_667_MOESM1_ESM.docx]

Supplementary Materials for

MG149 inhibits histone acetyltransferase KAT8-mediated IL-33 acetylation to alleviate allergic airway inflammation and airway hyperresponsiveness

Yahui Liu^1†^, Juan Du^1†^, Xinnan Liu^2^, Lingbiao Wang^3^, Yichao Han^4^, Chunrong Huang^1^, Rui Liang^2^, Fang Zheng^5^, Guochao Shi^1*^, Bin Li^2*^

Correspondence to: Guochao Shi (shiguochao@hotmail.com) or Bin Li (binli@shsmu.edu.cn)

**This PDF file includes:**

Materials and Methods

Figures. S1 to S12

Tables S1

**Materials and methods**

***Cell culture, transfection and drug treatment***

HEK293T (ATCC® CRL-11268™) cells, BEAS-2B cells (ATCC® CRL-9609™), and MLE12 cells were purchased from the American Type Culture Collection (Manassas, VA, USA) and cultured in Dulbecco's Modified Eagle Medium (DMEM, Gibco® 15140122 [Thermo Fisher Scientific, Waltham, MA, USA]) supplemented with 10% fetal bovine serum (FBS, Gibco® 16140071) and 1% Penicillin-Streptomycin (Gibco® 15140122). NHEB cells (Lonza® CC-2541 [Basel, Switzerland]) were cultured in BEGM (Lonza CC-3170) supplemented with 10% FBS (Gibco) and 1% Penicillin-Streptomycin (Gibco). HEK293T cells were transfected with polyethylenimine (PEI, Polysciences 23966-2 [Warrington, England]), following the manufacturer’s instructions.

For Trichostatin A (TSA), nicotinamide (NAM), or MG149 treatment to cell lines, stock solution was diluted to the final concentration using DMEM. Cells were treated with 1 μM TSA for 12 hours, 10 mM NAM for 6 hours, 20 μM MG132 for 6 hours and 1-10 μM MG149 for 12 hours. The same volume of solvent was used as a control.

For MG149 treatment *in vivo*, the working concentration was 0.2 mg/mL, and stock solution concentration was 4 mg/mL. Before each in vivo application, the stock solution was diluted with DMSO to the working concentration. Then, PEG300, Tween 80 and double distilled water were added in order according to the in vivo formulation calculator on the official website (https://www.selleckchem.com/). It was important to ensure that the solution obtained, in the previous addition, was a clear solution before proceeding to add the next solvent.

***Reagents and antibodies***

TSA (S1045) and MG149 (S7476) were purchased from Selleck (Houston, TX, USA). NAM (N0636) and MG132 (133407-82-6) were purchased from Merck (Kenilworth, NJ, USA). House dust mite (HDM) extract was purchased from Greer Laboratories (Boston, Massachusetts, USA). Proteinase inhibitor cocktail (P8340) was purchased from Sigma-Aldrich (Darmstadt, Germany). Ni-NTA beads (30210) for His pull-down assay was purchased from QAIGEN (Hilden, Germany). Protein A/G PLUS-Agarose (sc-2003) was purchased from Santa Cruz Biotechnology (Dallas, Texas, USA). Protein G Sepharose® was (P3296) purchased from Merck.

The following antibodies were used: anti-acetyl lysine antibody (9441, Cell Signaling Technology (CST), 1:1,000 for western blot, 1μg for immunoprecipitation), KAT8 antibody (ab200660, abcam, 1:1,000 for western blot, 1:500 for immunohistochemistry-paraffin), KAT8 antibody (sc-81163, Santa Cruz Biotechnology, 1:300 for western blot), monoclonal ANTI-Flag M2 antibody (F1804, Sigma-Aldrich, 1:10,000 for western blot, 1μg for immunoprecipitation), monoclonal ANTI-Flag M2-peroxidase (HRP) antibody (A8592, Sigma-Aldrich, 1:1,000 for western blot, 1μg for immunoprecipitation), V5-tag rabbit monoclonal antibody (13202, CST, 1:1,000 for western blot, 1μg for immunoprecipitation), β-tubulin rabbit monoclonal antibody (2128, CST, 1:1,000 for western blot), recombinant IL-33 antibody (ab207737, abcam, 1:1,000 for western blot, 1:1,000 for immunohistochemistry-paraffin, reacted with human IL-33), IL-33 antibody (ab54385, abcam, 1:1,000 for western blot, 1:1,000 for immunohistochemistry-paraffin, reacted with mouse IL-33), IL-33 antibody (AF3626, R&D systems, 0.4 µg/mL for western blot), IL-33 antibody (sc-517600, Santa Cruz Biotechnology, 1μg for immunoprecipitation), mouse anti-β-actin (60008, Proteintech, 1:10,000 for western blot), mouse anti-Myc (sc-40, Santa Cruz Biotechnology, 1:1,000 for western blot, 1μg for immunoprecipitation), rabbit IgG isotype (3900, CST, 1μg for immunoprecipitation), monoclonal ubiquitin antibody (3936, CST, 1:1,000 for western blot), rabbit anti-mouse IgG (light chain specific) monoclonal antibody (HRP Conjugate) (58802, CST, 1:1,000 for western blot), mouse anti-rabbit IgG (light-chain specific) monoclonal antibody (HRP Conjugate) (93702, CST, 1:1,000 for western blot), anti-mouse IgG-HRP conjugate (W4021, Promega, 1:5,000 for western blot), goat anti-rabbit IgG HRP-linked antibody (7074, CST 7074, 1:3,000 for western blot), goat anti-mouse IgG Alexa Fluor 488 (A11029, Thermo Fisher Scientific, 1:1,000 for immunofluorescence), goat anti-mouse IgG Alexa Fluor 555 (A32727, Thermo Fisher Scientific, 1:1,000 for immunofluorescence), goat anti-rabbit IgG Alexa Fluor 488 (A11008, Thermo Fisher Scientific, 1:1,000 for immunofluorescence), goat anti-rabbit IgG Alexa Fluor 555 (A27039, Thermo Fisher Scientific, 1:1,000 for immunofluorescence) and DAPI (4,6-diamidino-2-phenylindole, dihydrochloride, D1306, Invitrogen, 1:1,000 for immunofluorescence).

Mouse airway resistance activity was assessed by examining methacholine provocation via invasive lung function assessment (AniRes2005 V3.5, Animal Pulmonary Function Analysis System, Beijing Bestlab Technology Co., Ltd, China). Protein levels in bronchoalveolar lavage fluid (BALF) were measured using a Pierce™ BCA Protein Assay Kit (23225, Thermo Scientific). Total plasma immunoglobulin E (IgE) was measured using the BD OptEIA™ Set (555248, BD Bioscience [Franklin Lakes, NJ, USA]). Enzyme-linked immunosorbent assay (ELISA) was used to measure the levels of interleukin 4 (IL-4) (431104, Biolegend (San Diego, CA, USA)), IL-5 (431204, Biolegend), IL-13 (KMC2221, Invitrogen), IL-33 (DY3626, R&D DuoSet® ELISA DEVELOPMENT SYSTEM), and KAT8 (MBS288532, MyBioSource (San Diego, CA, USA)).

***Plasmids, lentivirus construction and infection***

Human IL-33 and KAT8 were amplified from NHEB cell cDNA and cloned into pcDNA 3.1 vectors with Flag, Myc, or V5 tags. A pcDNA3.1 V5-tagged KAT8 mutant K274R was constructed through site-directed mutagenesis and was confirmed via sequencing. PIP-2Flag-tagged KAT5 and PIP-6his-tagged Ubiquitin were constructed as described previously.^1^ Truncations of IL-33 and KAT8 were generated by polymerase chain reaction (PCR)-based amplification and confirmed by sequencing. Mouse IL-33 and KAT8 were amplified from MLE12 cell (CRL-2100) cDNA and were cloned into pcDNA 3.1 vectors.

KAT8 knockout cells were generated by lentiCRISPR system. CRISPRs were designed using a CRISPR design web tool (http://crispr.mit.edu). The sgRNA (single guide RNA) sequences targeted by KAT8 is AACGTACCTGTGCCGGCGACCGG. A monoclonal KAT8 knockout HEK293T cell line was generated by limiting dilution.

***Mice***

*Il33^-/-^* C57BL/6 mice were provided by Professor Fang Zheng. All animal experiments were performed in accordance with the National Institutes of Health *Guide for the Care and Use of Laboratory Animals* and with the approval of the Institutional Animal Care and Use Committee in Institute Pasteur of Shanghai. All mice were maintained under specific pathogen-free conditions. Female 6- to 8- week-old mice were used for experiments. Randomization and blinding strategies were used whenever possible. For the allergic asthma model, mice were challenged for five consecutive days in two weeks with intranasal administration of 35 μL (0.7 mg/mL phosphate buffered saline (PBS)) of whole HDM protein extract. In the HDM + MG149 group, the anaesthetized mice were pretreated with intraperitoneal injection of MG149 (1 mg/kg/d) 60 minutes prior to HDM challenge. Control animals received only PBS. All mice were sacrificed by overdose of pentobarbital 24 hours after the last challenge.^2^ All experiments were repeated at least three times with similar sample sizes.

***Clinical samples***

Three specimens from asthma patients and four surgical specimens of lobectomy or segmentectomy patients were obtained with the approval of the Institutional Review Board and Human Ethics Committee of Ruijin Hospital, Shanghai Jiao Tong University School of Medicine. All the subjects provided written informed consent. The three enrolled asthma patients were diagnosed with asthma according to the *Global Initiative for Asthma (2020 edition)*.^3^ All surgical specimens were taken from the peri-tumoral tissues of patients without asthma. The demographic details of the enrolled patients are in Supplemental Table S1.

***Immunoprecipitation and western blot***

Cells were harvested, washed with PBS and lysed with radio immunoprecipitation assay buffer (RIPA buffer) (0.5% NP40, 150 mM NaCl, 50 mM Tris-HCl, PH 7.5), which was supplemented with 1% proteinase inhibitor cocktail, 1 mM PMSF, 1 mM NaF, and 1 mM Na_3_VO_4_. Cell lysates were then sonicated and centrifugated at 12,000 × *g* for 15 min at 4℃. The supernatants were separated by SDS-PAGE and analyzed by immunoblotting. Proteins were visualized using enhanced chemiluminescence (WBKLS0500, Millipore).

To detect IL-33 acetylation and the interaction between IL-33 and KAT8, cells were lysed in RIPA buffer supplemented with 1% proteinase inhibitor cocktail, 1 mM PMSF, 1 mM NaF, 1 mM Na_3_VO_4_, 1 μM TSA and 10mM NAM. The supernatants were immunoprecipitated with Flag antibody, V5 antibody, IL-33 antibody, anti-acetyl lysine antibody, and KAT8 antibody for 1 hour at 4℃. After that, the immunoprecipitants were washed six times with lysis buffer and boiled in 1 × SDS-loading buffer for immunoblot analysis. For immunoprecipitation and western blot of mouse lung tissue, the homogenate was diluted 8~10 times before testing.

**Flow cytometry**

Single cells were incubated with anti-mouse CD16 and CD32 (93, TruStain fcX, BioLegend) at a 1:100 dilution to block Fc receptors. BD Horizon^TM^ Fixable Viability Stain 510 (564406) or BD Horizon^TM^ Fixable Viability Stain 570 (564995) was used to exclude dead cells, used according to the manufacturer’s instructions. The following murine antibodies were used for flow cytometry: biotinylated anti-mouse lineage CD3ε (145-2C11), CD5 (53-7.3), TCRβ (H57-597), TCR-γδ (eBioGL3), TCRb (H57-597), CD45R (RA3-6B2), Gr-1 (RB6-8C5), CD11c (N418), CD11b (M1/70), Ter119 (TER-119), FcεRIα (MAR-1), Streptavidin-FITC, PE/Cyanine7 anti-mouse CD127 (IL-7Rα) antibody (A7R34, Biolegend), APC/Cyanine7 anti-mouse CD45 antibody (30-F11, Biolegend), FITC anti-mouse CD3 antibody (17A2, Biolegend), and PerCPCy5.5 anti-mouse CD3 (17A2, Biolegend). Intracellular staining was performed using the BD Cytofix/Cytoperm kit. When indicated, cytokine production was measured following 4 hours in vitro stimulation with 50μg/mL PMA (Sigma), 500μg/mL ionomycin (Sigma) and/or 1μg/mL Golgi plug (BD Bioscience). Brilliant Violet 421™ anti-GATA3 antibody (16E10A23, Biolegend), PE anti-mouse/human IL-5 antibody (TRFK5, Biolegend), PE anti-mouse IL-13 antibody (W17010B, Biolegend), and PE-Cyanine7 IL-13 monoclonal antibody (eBio13A, eBioscience) were used. Stained cells were analyzed on FACSCanto II and/or FACSARIA III systems and the data was analyzed with FlowJo version 10 software.

***His-ubiquitin pulldown assay***

His-ubiquitin pulldown assay was performed as described previously.^4^ Cells were harvested, washed with PBS, and lysed in a pH 8.0 urea buffer (8 M urea, 100 mM Na_2_HPO_4_, 10 mM Tris-HCl (pH 8.0), 0.2% Triton X-100, 10 mM imidazole, and 1 mM N-ethylmaleimide). Next, they were incubated with Ni-NTA Beads for 3 hours at room temperature. The beads were washed twice in pH 8 urea buffer, twice in pH 6.3 urea buffer (8 M urea, 100 mM Na_2_HPO_4_, 10 mM Tris-HCl (pH 6.3), 0.2% Triton X-100, and 10 mM imidazole), and once in a wash buffer (20 mM Tris-HCl (pH 8.0), 100 mM NaCl, 20% glycerol, 1 mM dithiothreitol, and 10 mM imidazole). Samples were then used for immunoblotting analysis with indicated antibodies.

***Measurement of airway resistance***

Airway resistance was assessed by methacholine provocation testing. Respiratory mechanics were recorded using invasive lung function assessment. In brief, the mice were intraperitoneally anesthetized with pentobarbital. The cannula of the trachea was connected to a pressure transducer to measure transpulmonary pressure. Airflow and tidal volume were determined using a flow transducer. The mice were ventilated with an average breathing frequency of 150 breaths/min, tidal volume of 10 ml/kg body weight, and positive end-expiratory pressure of about 2 cm H_2_O. For methacholine (MCh) challenge, ∼35 µL of PBS containing from 0 to 100 mg/ml MCh was injected into the mice via the external jugular vein, and pulmonary resistance (RL) was measured for 5 min in each dose. RL was calculated by dividing transpulmonary pressure by airflow. The percentage of RL change was presented in the results.

***Sample collection and preparation***

Mice were anesthetized with an overdose of sodium pentobarbital followed by exsanguinating mice from the vena inguinalis to collect the blood, which was used to measure plasma total IgE. After the tracheotomy, BALF was collected by flushing the lungs three times with 1 mL cold PBS-ethylene diamine tetra acetic acid (EDTA). The supernatant was collected after centrifuging the BALF, followed by measuring the levels of total protein using a BCA protein assay kit, and the cell pellet was resuspended, and used for cell counts and Wright-Gimsa staining. Differential cell counts of BALF were determined by counting a minimum of 300 cells/slide using standard morphological criteria in a single-blind method. The mediastinal lymph nodes (mLNs) were made into a single cell suspension, and stimulated with HDM (30 μg/mL). The supernatants were used to perform ELISA to detect type 2 cytokines. Murine lungs harvested without bronchoalveolar lavage were ground into lung homogenate to detect type 2 cytokine expression, and also underwent RNA extraction and histological staining.

***Quantitative real-time PCR***

Total RNA was extracted from mouse lungs using TRIzol. RNA was quantified and complementary DNA was reverse transcribed with 1 μgRNA using the PrimeScript RT Reagent Kit (Takara, RR037A). PCR reactions were run in an ABI Prism 7500 Sequence Detection System. Quantification of relative mRNA expression was determined by the comparative CT method. Relative gene expression was determined via normalization to the housekeeping gene β-actin. Primers are as follows: *β-actin* forward, GGCTGTATTCCCCTCCATCG; *β-actin* reverse, CCAGTTGGTAACAATGCCATGT; *Il4* forward, ttgagagagatcatcggcattttg; *Il4* reverse, tcaagcatggagttttcccatgt; *Il5* forward, tgttgacaagcaatgagacgatga; *Il5* reverse, aatagcatttccacagtaccccca; *Il13* forward, CGGCAGCATGGTATGGAGTGTG; *Il13* reverse, GGAGGCTGGAGACCGTAGTGG; *Il33* forward, ctggcctcaccataagaaaggaga; *Il33* reverse, agggaggcaggagactgtgttaaa; *Kat8* forward, acgaggcgatcaccaaagtg; *Kat8* reverse, aagcggtagctcttctcgaac.

***ELISA***

Lung tissue was homogenized at 50 mg/mL in Hank’s balanced salt solution containing protease inhibitor tablets (Roche Diagnostics) and centrifuged at 12000*g* for 20 min, and the supernatant was collected for quantification for targeted proteins. IL-4 (431104, Biolegend), IL-5 (431204, Biolegend), IL-13 (KMC2221, Invitrogen), IL-33 (DY3626, R&D DuoSet® ELISA DEVELOPMENT SYSTEM), and KAT8 (MBS288532, MyBioSource (San Diego, CA, USA)) were used in standardized sandwich enzyme-linked immunosorbent assay according to the manufacturer’s protocols.

***Histology and confocal immunofluorescence***

Hematoxylin and eosin (HE) and periodic acid-Schiff (PAS) staining was performed as described previously.^5^ Mouse lung tissues and human specimens were fixed in 4% formalin and cut into 5-μm sections. Sections were dewaxed and rehydrated and used for HE, PAS, and Masson’s Trichrome staining. Quantitative analysis for HE, PAS, and Masson’s Trichrome staining was performed as described previously.^6,7^

Immunohistochemistry was performed as described previously.^5^ Mouse lung tissue sections were incubated with anti-IL-33 antibody overnight at 4°C. Peroxidase-conjugated goat anti-rabbit IgG was then used for one hour at 37°C, followed by addition of the streptavidin-HRP conjugate and substrate chromogen mixture. Finally, all slides were counterstained with hematoxylin.

For the immunofluorescence (IF) staining, tissue sections were incubated with IL-33 antibody and KAT8 antibody after antigen retrieval and blocked with 2% horse serum in PBS. The secondary antibodies used were as mentioned above. Nuclei were counterstained with DAPI (4′,6-diamidin-2-fenilindolo). A Zeiss LSM confocal microscope was used to acquire images that were generated by collecting consecutive scans with one laser line active per scan to prevent excitation crosstalk. Quantitative analysis in lung tissues was performed using ImageJ.

***Statistical analysis***

Each experiment was performed at least three times. The significant differences in statistical data were determined by two-tailed, unpaired Student’s t-tests using GraphPad Prism 8. All data are expressed as means ± standard error of the mean (SEM). Unless otherwise indicated, the data are derived from more than three independent experiments. A *P* value < 0.05 was considered to indicate statistical significance.

**References**

1. Chen, Z. *et al.* The ubiquitin ligase stub1 negatively modulates regulatory T cell suppressive activity by promoting degradation of the transcription factor Foxp3. *Immunity* **39**, 272–285 (2013).

2. Piyadasa, H. *et al.* Biosignature for airway inflammation in a house dust mite-challenged murine model of allergic asthma. *Biol. Open* **5**, 112–121 (2016).

3. GINA Executive and Science committee. Global Initiative for Asthma: Global strategy for asthma management and prevention (Updated 2020). https://ginasthma.org/wp-content/uploads/2020/04/GINA-2020-full-report_-final-_wms.pdf doi:10.1016/S0335-7457(96)80056-6.

4. Li, Y. *et al.* USP21 prevents the generation of T-helper-1-like Treg cells. *Nat. Commun.* **7**, 1–10 (2016).

5. Liu, T. *et al.* Autophagy plays a role in FSTL1-induced epithelial mesenchymal transition and airway remodeling in asthma. *Am. J. Physiol. - Lung Cell. Mol. Physiol.* **313**, L27–L40 (2017).

6. Myou, S. *et al*. Blockade of inflammation and airway hyperresponsiveness in immune-sensitized mice by dominant-negative phosphoinositide 3-kinase-TAT. J Exp Med. 198(10):1573-82 (2003).

7. Marcos-Garcés, V. *et al*. Comparative measurement of collagen bundle orientation by Fourier analysis and semiquantitative evaluation: reliability and agreement in Masson's trichrome, Picrosirius red and confocal microscopy techniques. J Microsc. 267(2):130-142 (2017).

**Figure. S1.**


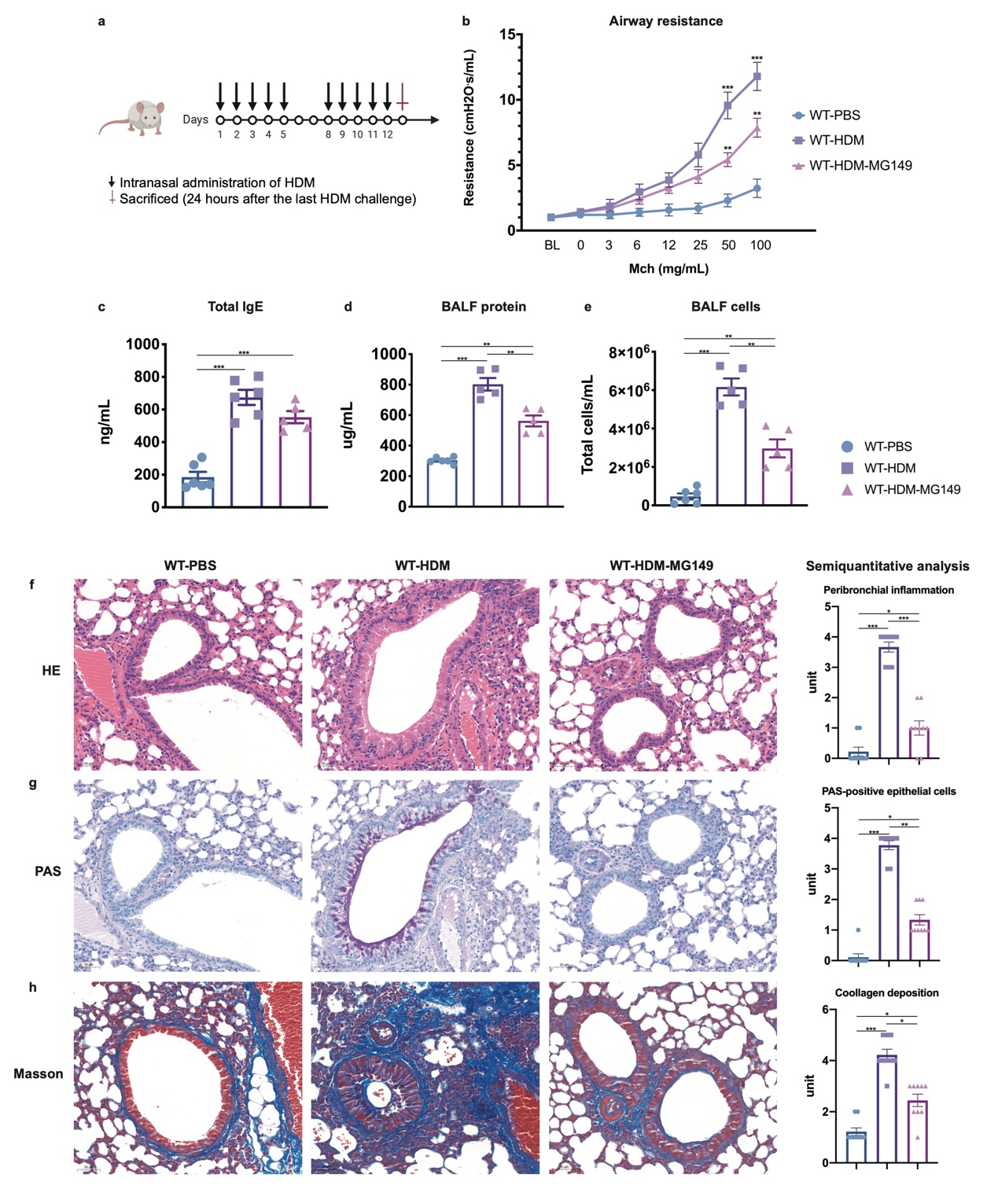


**Figure. S1** **a** Schematic diagram of house dust mite (HDM) induced allergic asthma model. **b** Airway responsiveness to methacholine (Mch) in wild-type (WT) mice exposed to intranasal PBS or HDM and intraperitoneal MG149 was measured. **c** Levels of total plasma IgE. **d** Total protein levels in bronchoalveolar lavage fluid (BALF). **e** Total cell numbers in BALF. **f** HE staining of mouse lung tissues. **g** PAS staining of mouse lung tissues. **h** Masson staining of mouse lung tissues. Error bars represent standard error of the mean (SEM). Data are mean ± SEM. Individual values are shown for n = 5~6 mice per treatment. Comparisons were made using unpaired t test. Data were from three independent experiments. **P* < 0.05, ***P* < 0.01, ****P* < 0.001.

**Figure. S2.**


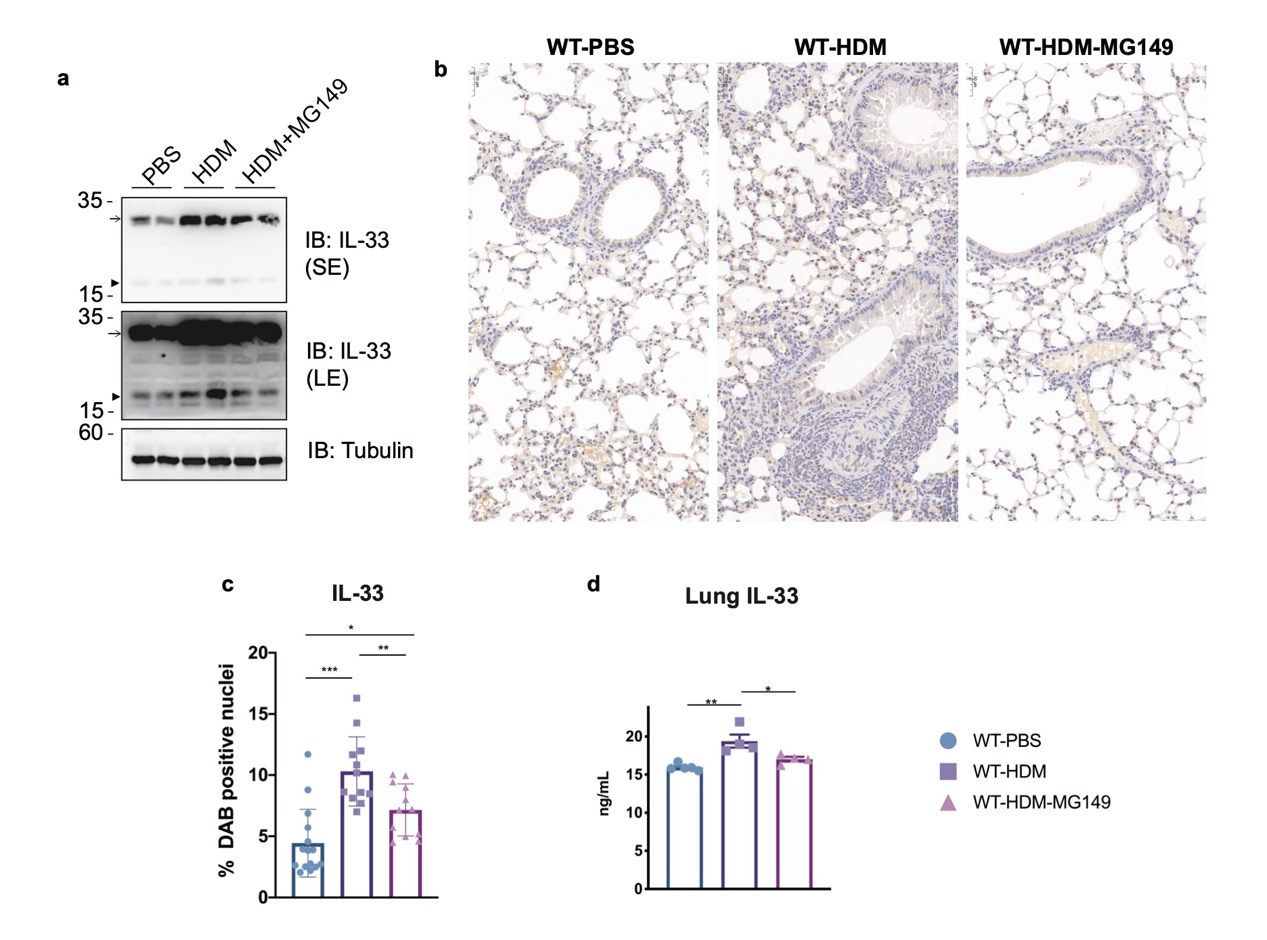


**Figure. S2 a** IL-33 levels in mouse lung homogenate were detected by western blot. **b** Immunohistochemistry (IHC) of IL-33 in lung sections from WT mice. **c** Semi-quantitative analysis of IL-33 of WT mice by IHC staining. **d** IL-33 levels in WT mice lung homogenate were measured by ELISA. Error bars represent SEM. Data are mean ± SEM. Individual values are shown for n = 4~5 mice per treatment. Comparisons were made using unpaired t test. Data were from three independent experiments. **P* < 0.05, ***P* < 0.01, ****P* < 0.001. →, full length IL-33; , cleaved IL-33.

**Figure. S3.**


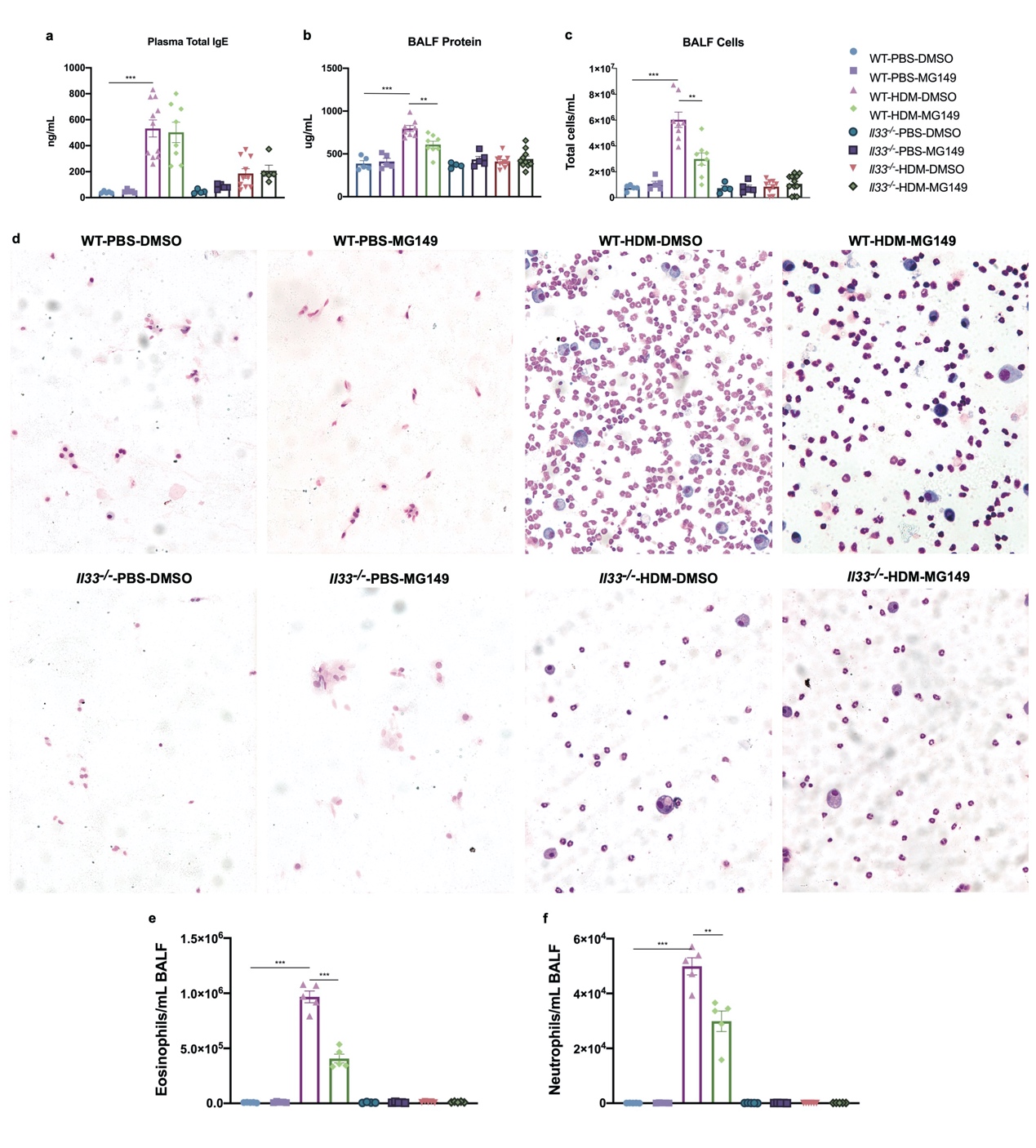


**Figure. S3** **a** Levels of total plasma IgE in WT mice and *Il33* knockout mice exposed to intranasal PBS or HDM and intraperitoneal DMSO or MG149 were measured. **b** Total protein levels in BALF were assessed by BCA kit. **c** Total cell numbers in BALF. **d** Representative images of Wright-Gimsa staining of cells in BALF. **e** The changes of eosinophils in BALF of WT mice and *Il33* knockout mice exposed to intranasal PBS or HDM and intraperitoneal DMSO or MG149. **f** The changes of neutrophils in BALF of WT mice and *Il33* knockout mice exposed to intranasal PBS or HDM and intraperitoneal DMSO or MG149. Error bars represent SEM. Data are mean ± SEM. Individual values are shown for n = 4~12 mice per genotype and treatment. Comparisons were made using unpaired t test. Data were from three independent experiments. *, P < 0.05, **, P < 0.01, ***, P < 0.001.

**Figure. S4.**


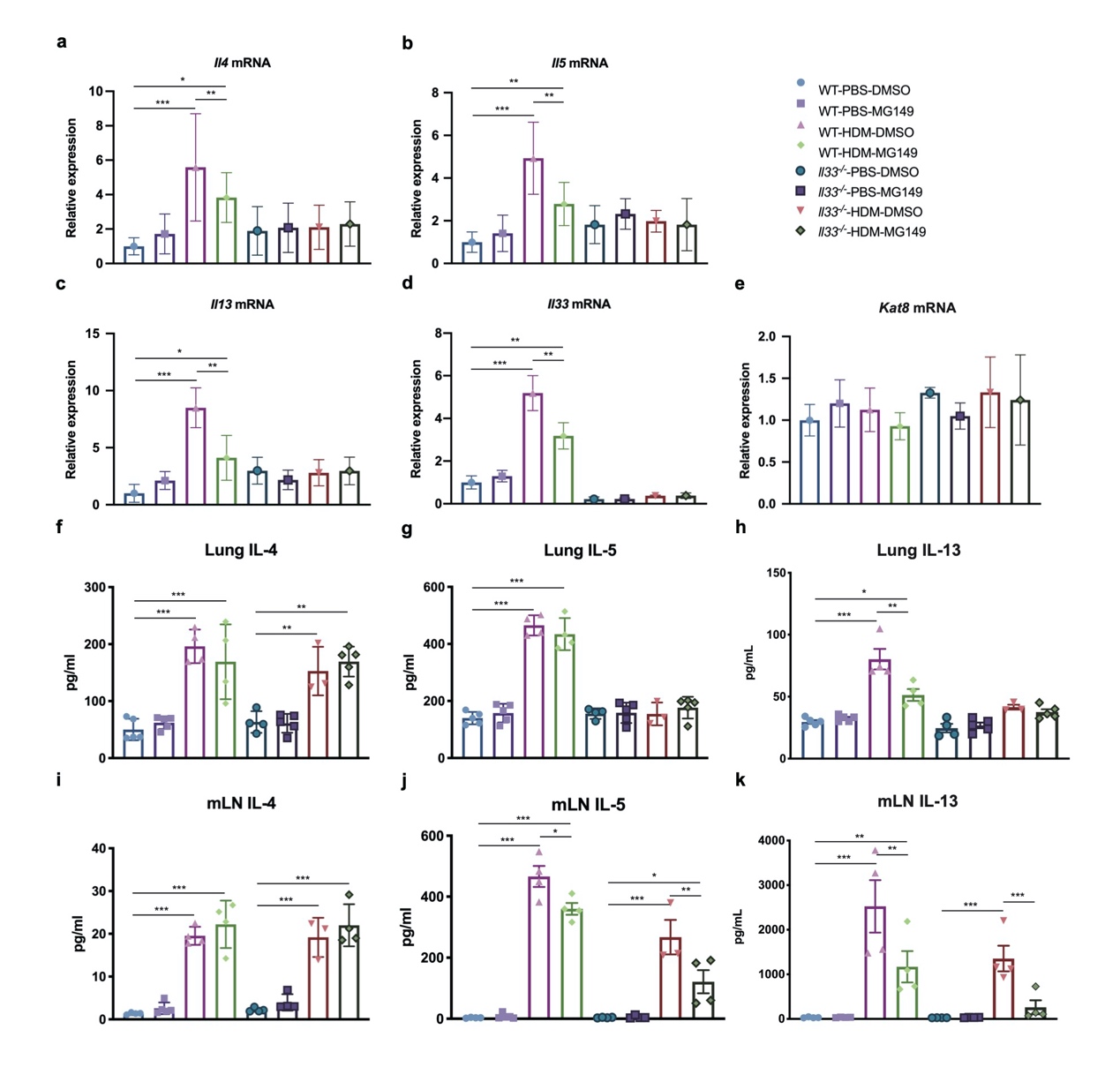


**Figure. S4** Expression of *Il4* (**a**), *Il15* (**b**), *Il13* (**c**), *Il33* (**d**), and *Kat8* (**e**) mRNA of mouse lung tissues. IL-4 (**f**), IL-5 (**g**), and IL-13 (**h**) protein levels in lung homogenate were assessed by ELISA. IL-4 (**i**), IL-5 (**j**), and IL-13 (**k**) protein levels in mediastinal lymph nodes (mLNs) were assessed by ELISA. Error bars represent SEM. Data are mean ± SEM. Individual values are shown for n = 3~5 mice per genotype and treatment. Comparisons were made using unpaired t test. Data were from three independent experiments. *, P < 0.05, **, P < 0.01, ***, P < 0.001.

**Figure. S5.**


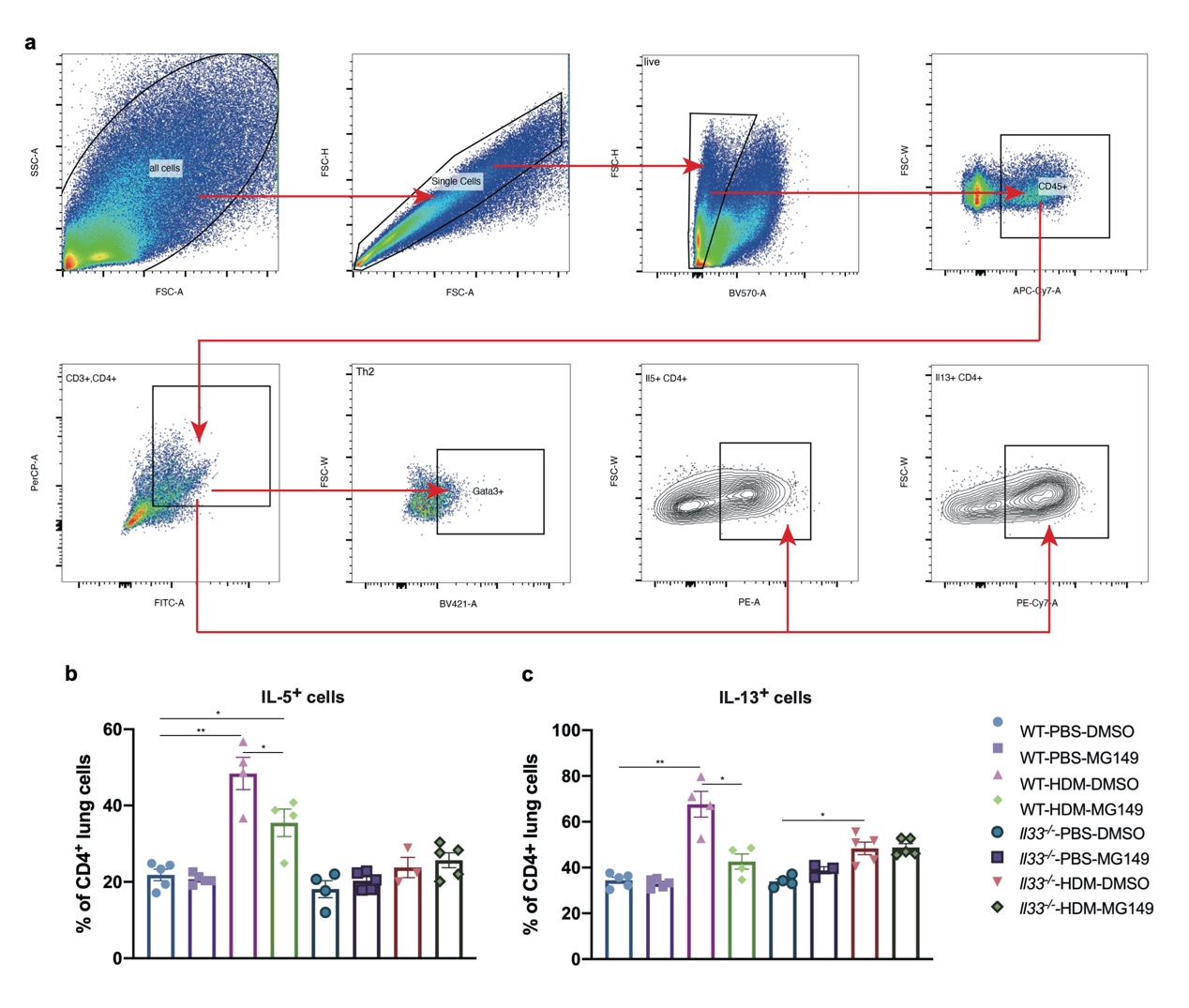


**Figure. S5 a** Hierarchical gating strategy for Th2 cells, IL-5^+^ CD4^+^ cells, and IL-13^+^ CD4^+^ cells, defined as singlet, live, CD45, CD4, CD3 cells with positive intracellular GATA3, IL-5 or IL-13 staining were then selected. **b** Percentages of IL-5^+^ CD4^+^ cells in lung tissues by flow cytometry. **c** Percentages of IL-13^+^ CD4^+^ cells in lung tissues by flow cytometry. Error bars represent SEM. Data are mean ± SEM. Individual values are shown for n = 3~5 mice per genotype and treatment. Comparisons were made using unpaired t test. Data were from three independent experiments. *, P < 0.05, **, P < 0.01, ***, P < 0.001.

**Figure. S6.**


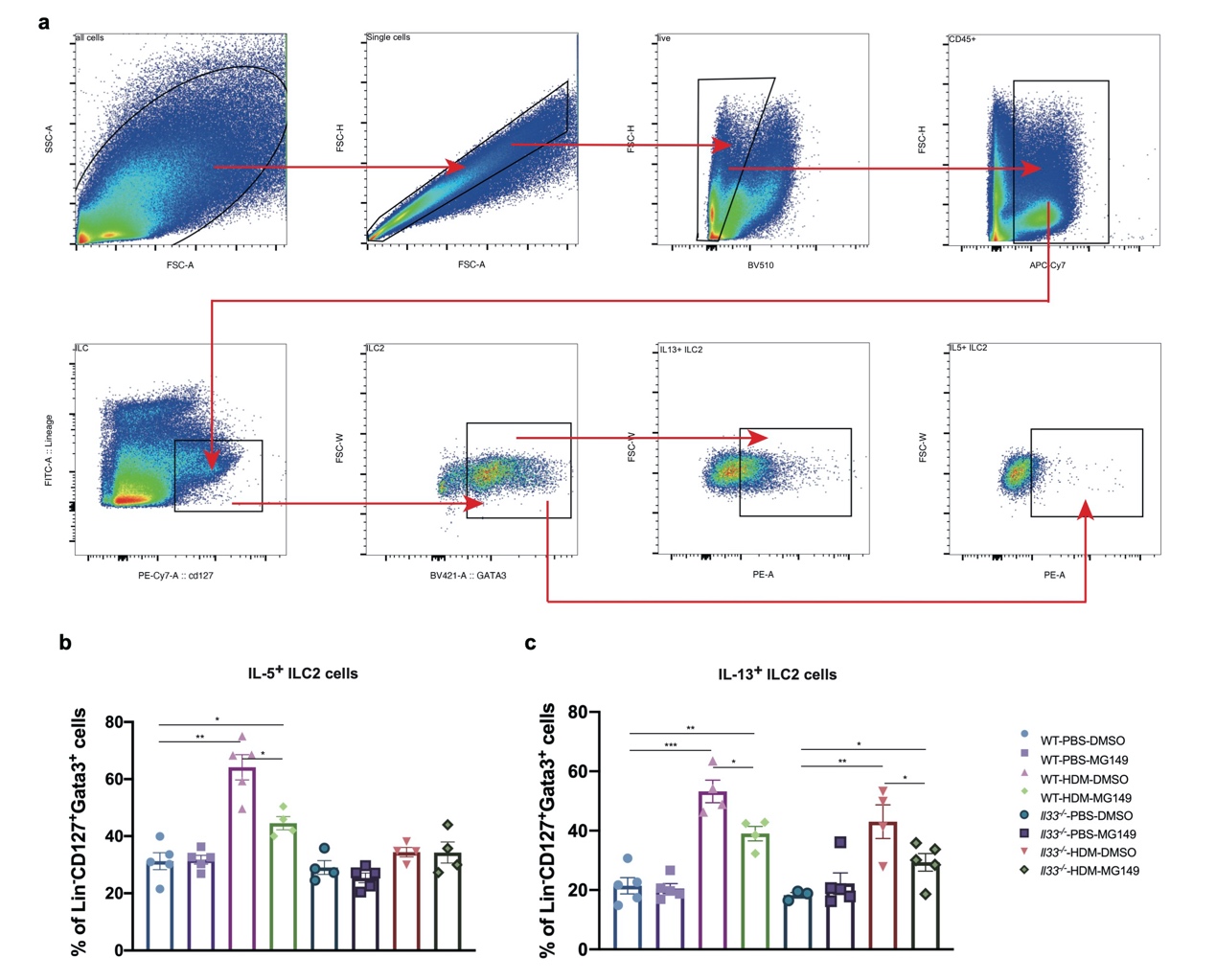


**Figure. S6 a** Hierarchical gating strategy for ILC2, IL-5^+^ ILC2 cells, and IL-13^+^ ILC2 cells, defined as singlet, live, CD45, CD127, Lin^-^ cells with positive intracellular GATA3, IL-5 or IL-13 staining were then selected. **b** Percentages of IL-5^+^ ILC2 cells in lung tissues by flow cytometry. **c** Percentages of IL-13^+^ ILC2 cells in lung tissues by flow cytometry. Error bars represent SEM. Data are mean ± SEM. Individual values are shown for n = 3~5 mice per genotype and treatment. Comparisons were made using unpaired t test. Data were from three independent experiments. *, P < 0.05, **, P < 0.01, ***, P < 0.001.

**Figure. S7.**


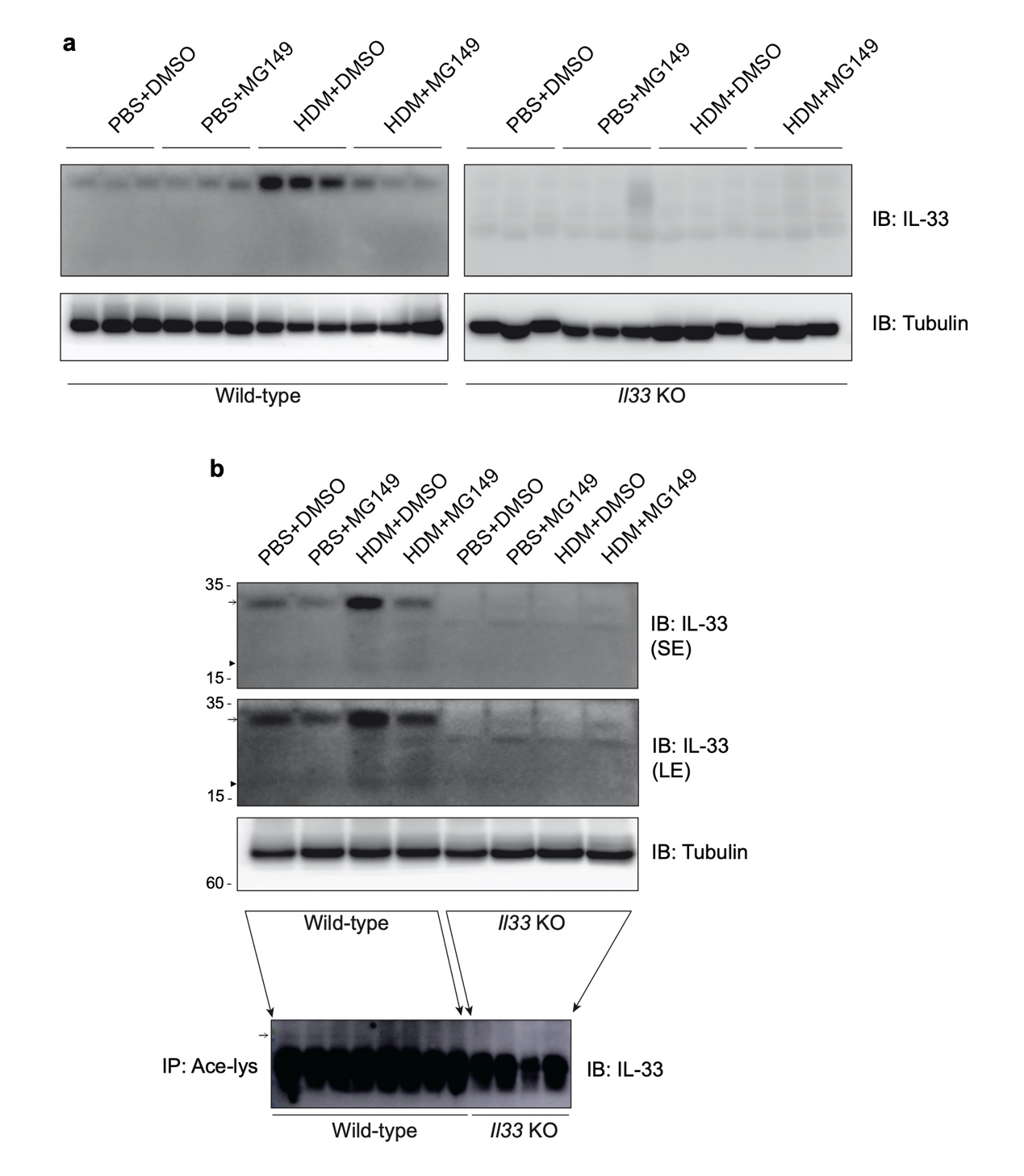


**Figure. S7 a** IL-33 levels in mouse lung homogenate were detected by western blot. **b** Immunoprecipitation of endogenous IL-33 using antibodies against anti-acetyl lysine antibody (Ace-lys) in mouse lung homogenate. →, full length IL-33.

**Figure. S8.**


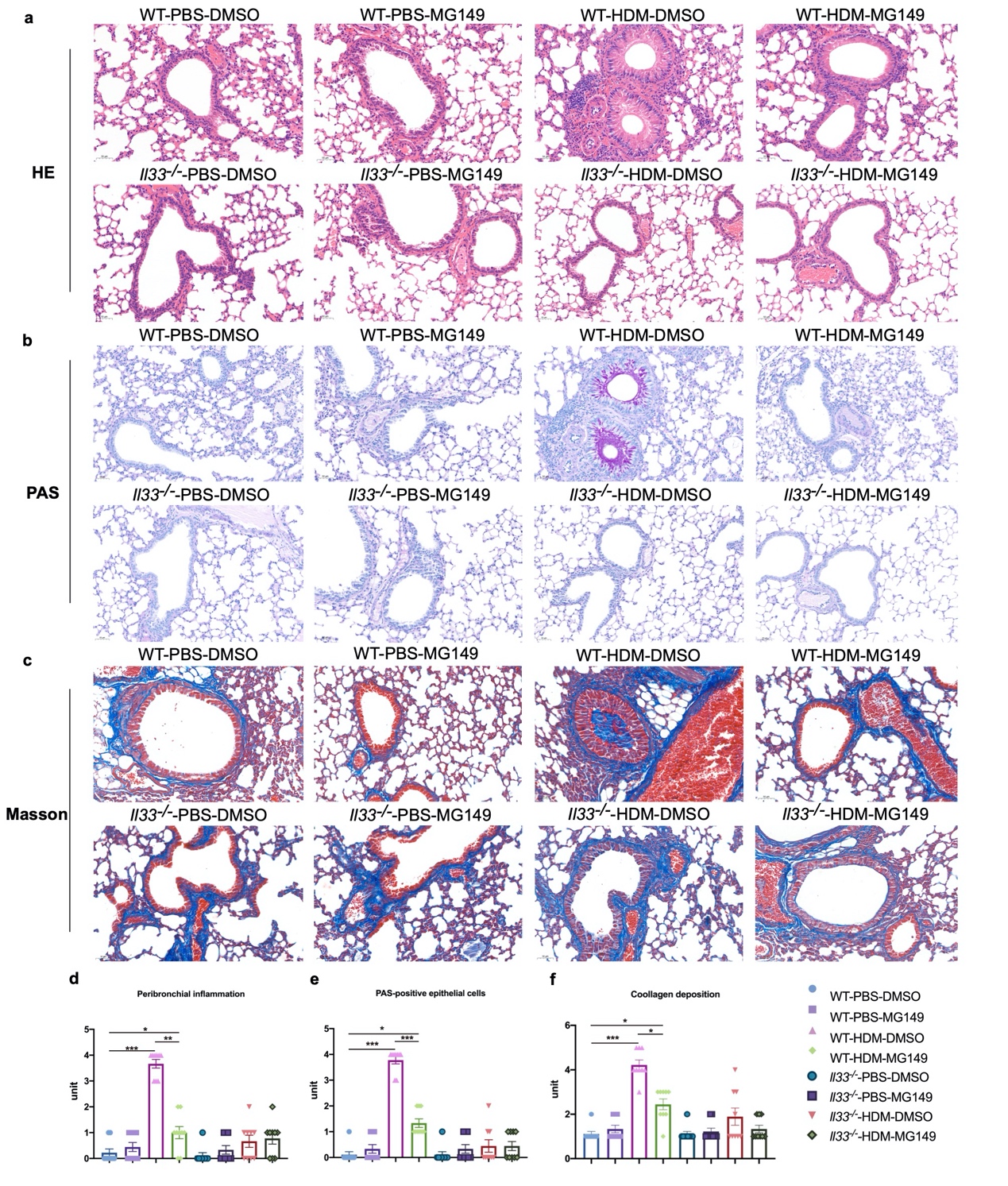


**Figure. S8** **a** HE staining of mouse lung tissues. **b** PAS staining of mouse lung tissues. **c** Masson staining of mouse lung tissues. Assessment of lung inflammation and airway remodeling via semi-quantitative analysis of HE staining (**d**), PAS staining (**e**), and Masson staining (**f**). Error bars represent SEM. Data are mean ± SEM. Individual values are shown for n = 3~4 mice per genotype and treatment. Comparisons were made using unpaired t test. Data were from three independent experiments. **P* < 0.05, ***P* < 0.01, ****P* < 0.001.

**Figure. S9.**


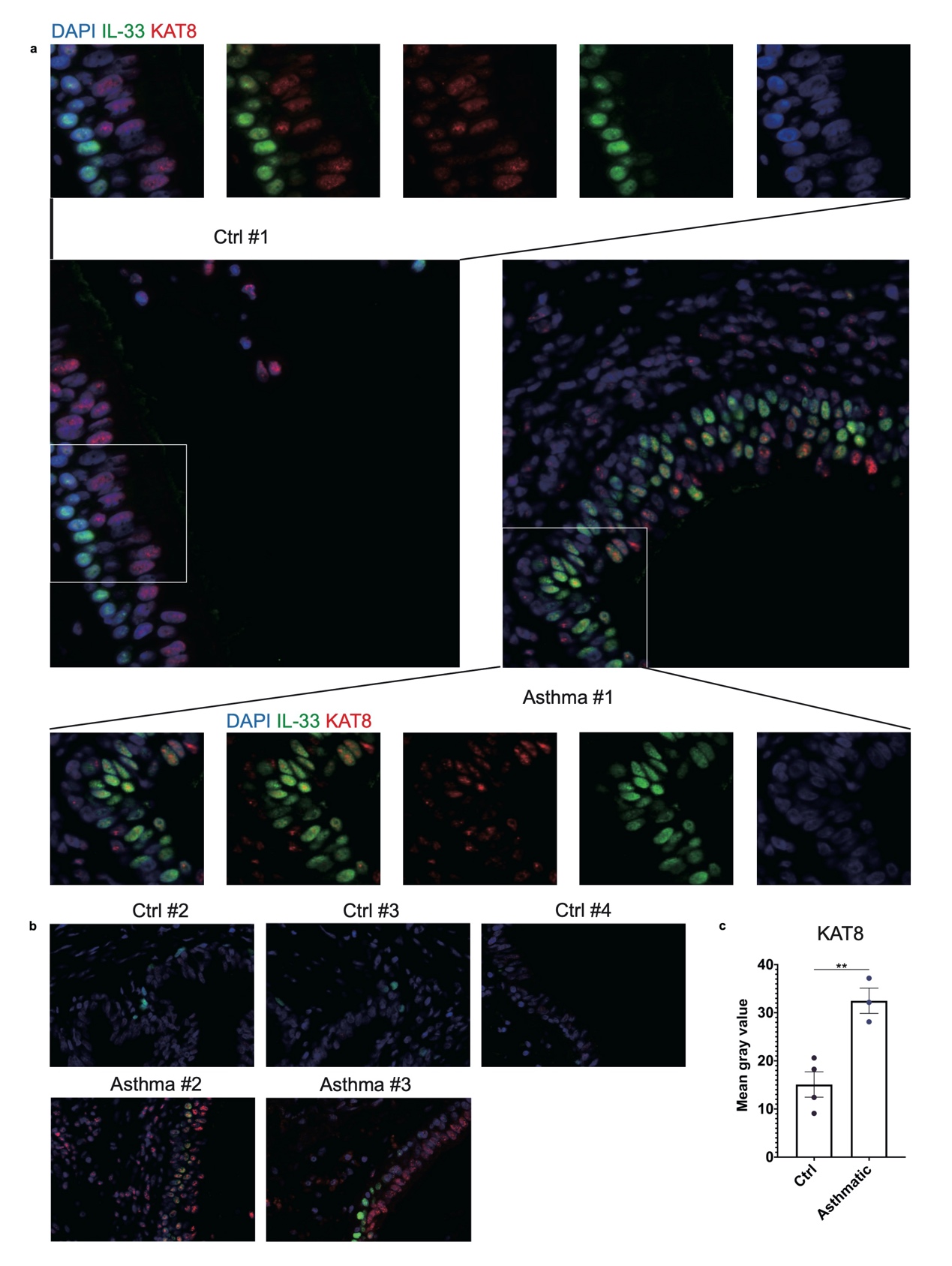


**Figure. S9** **a** Representative confocal images of lung tissues from asthma patients and controls labeled for IL-33 (green), and KAT8 (red). The nuclei were stained with DAPI (blue). 600 ×. **b** Representative confocal images of other three specimens from surgical specimens of lobectomy or segmentectomy patients and two specimens from asthma patients. **c** Semi-quantitative analysis of KAT8 in the controls and asthma patients via IF staining. Data are mean ± SEM. Comparisons were made using unpaired t test. ***P* < 0.01.

**Figure. S10.**


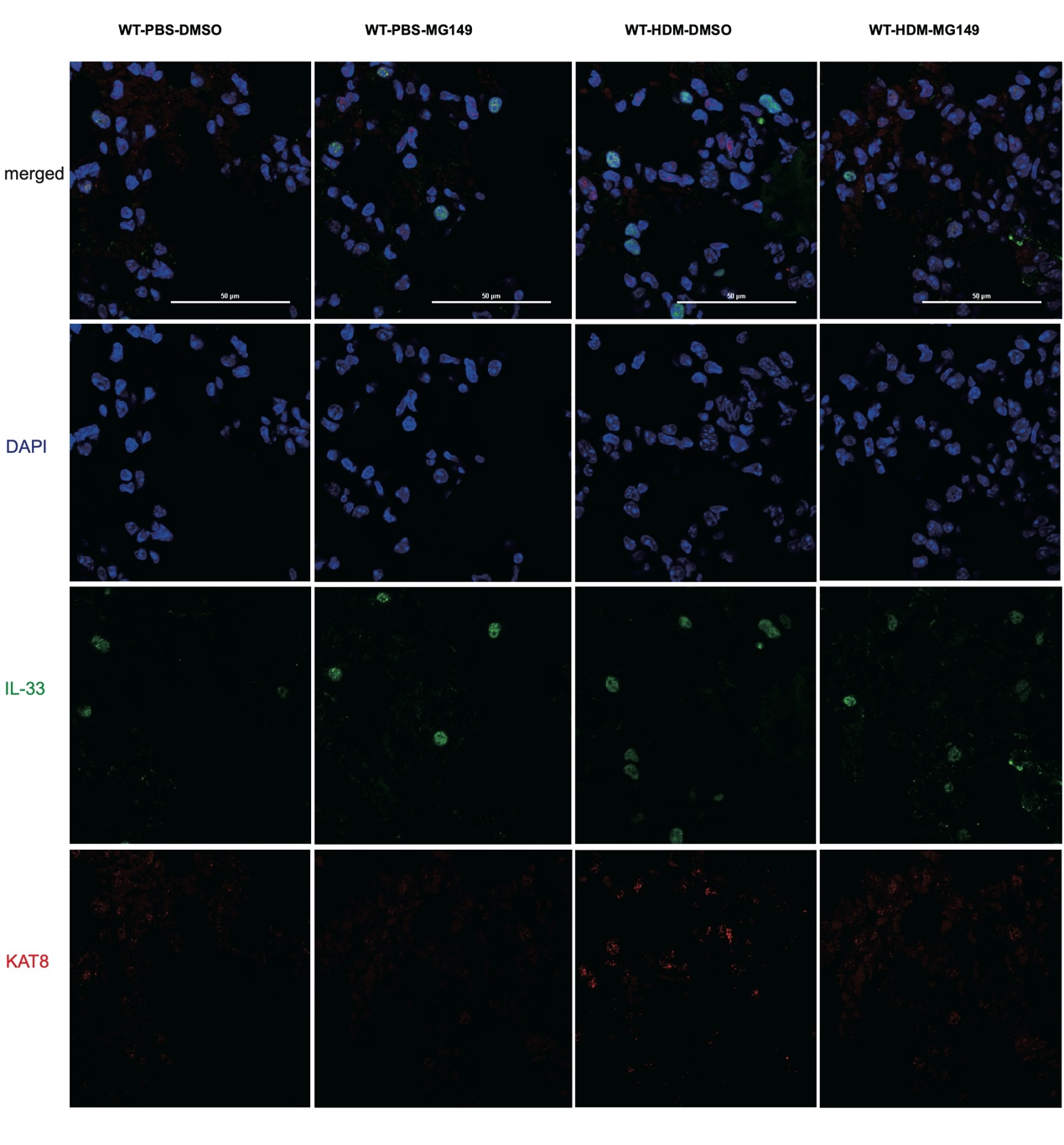


**Figure. S10** Representative confocal images of IF staining of lung sections from mouse labeled for IL-33 (green), and KAT8 (red). The nuclei were stained with DAPI (blue). 600 ×.

**Figure. S11.**


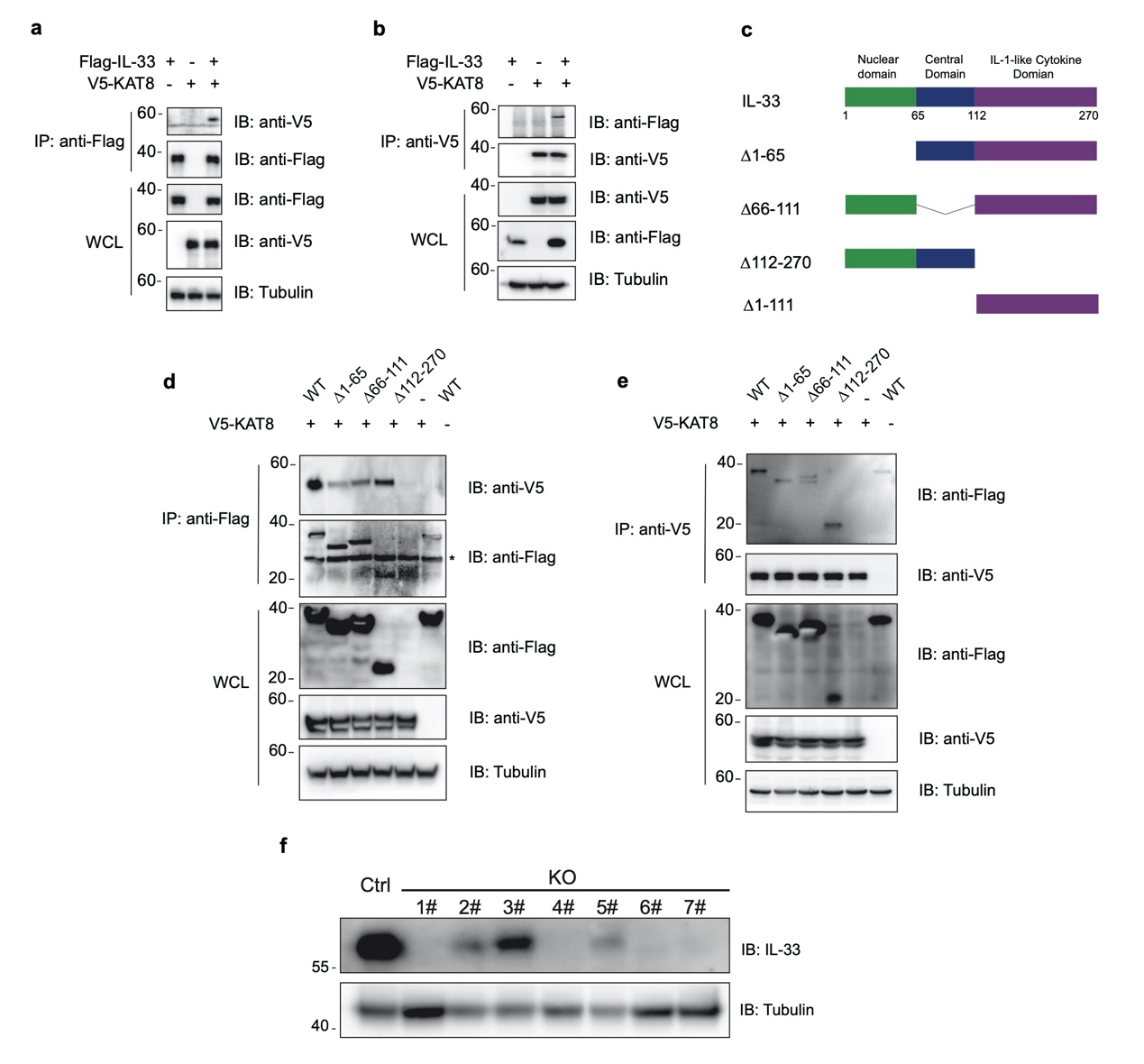


**Figure. S11** **a** Co-immunoprecipitation of exogenous mouse IL-33 and KAT8 in HEK293T cells via cell lysates immunoprecipitated with anti-Flag. **b** Co-immunoprecipitation of exogenous human IL-33 and KAT8 in HEK293T cells via cell lysates immunoprecipitated with anti-V5. **c** Schematic representation of Flag-tagged human IL-33 truncations. Co-immunoprecipitation analysis for the interaction between full-length KAT8 and different truncations of IL-33 via immunoprecipitation with anti-Flag (**d**) and anti-V5 (**e**). **f** Western blot screening of KAT8 knockout monoclonal HEK293T cell line. *, IgG light chain.

**Figure. S12.**


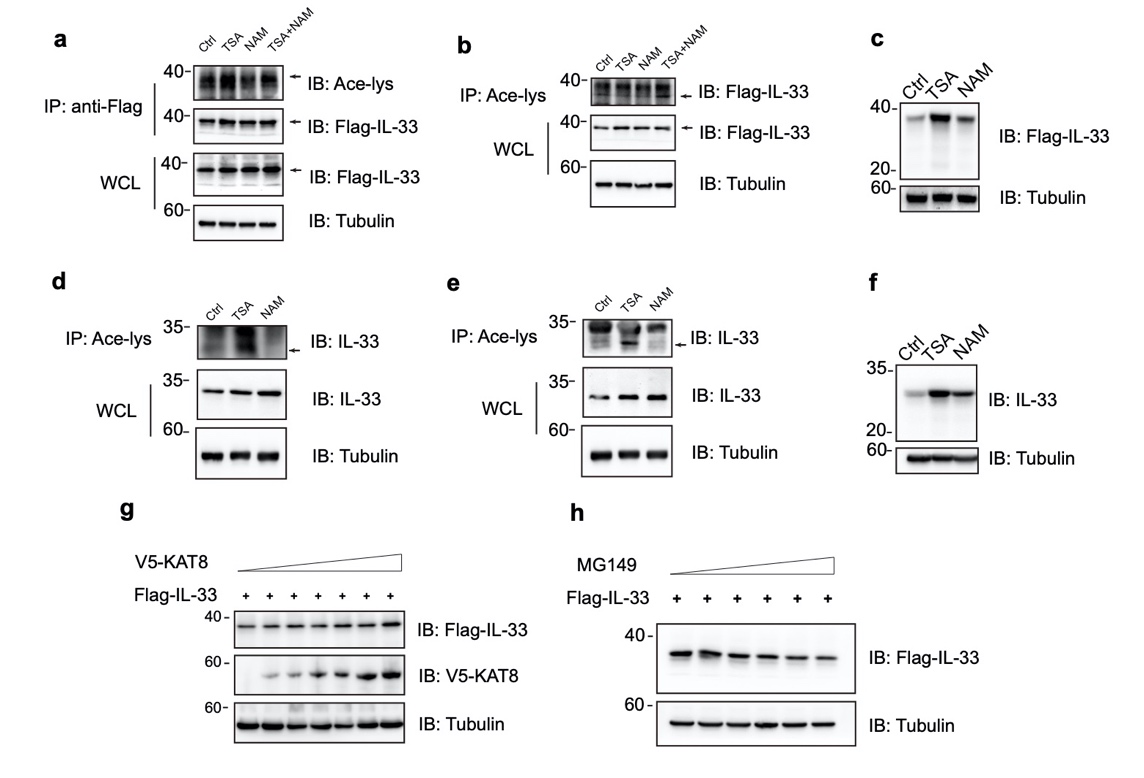


**Figure. S12** **a** Acetylation of exogenous Flag-IL-33 in HEK293T cells treated with TSA or NAM. Anti-Flag was immunoprecipitated with Flag-IL-33. The precipitates were analyzed with anti-acetyl lysine antibody (Ace-lys). **b** Acetylation of exogenous Flag-IL-33 in HEK293T cells treated with TSA or NAM. IL-33 acetylation was analyzed through immunoprecipitating with anti-acetyl lysine antibody followed by western blotting for Flag-IL-33. Acetylation of endogenous IL-33 in NHBE cells (**d**) and BEAS-2B cells (**e**) treated with TSA or NAM was analyzed via immunoprecipitation with anti-acetyl lysine antibody followed by western blotting for IL-33. TSA and NAM treatment increased exogenous IL-33 levels in HEK293T cells (**c**) and endogenous IL-33 in BEAS-2B cells (**f**). **g** KAT8 overexpression increased exogenous IL-33 levels in HEK293T cells, which was dose-dependent. Inhibition of endogenous KAT8 via adding MG149 (1-10 μM) reduced exogenous IL-33 levels in HEK293T cells (**h**).

**Table S1. Characteristics of the enrolled asthma patients and lung cancer patients**

| Case No. | Gender | Age | Diagnosis | Assessment of asthma | Cancer stage | Treatment history |
| --- | --- | --- | --- | --- | --- | --- |
| #1 | Female | 59 | Bronchial asthma | Well controlled | / | As-needed low dose ICS-formoterol |
| #2 | Male | 47 | Bronchial asthma | Well controlled | / | Low dose ICS-formoterol |
| #3 | Female | 38 | Bronchial asthma | Partly controlled | / | Low dose ICS-LABA |
| #4 | Female | 67 | NSCLC | / | T1N0M0 | Captopril for hypertension |
| #5 | Female | 54 | NSCLC | / | T1N0M0 | No |
| #6 | Male | 46 | NSCLC | / | T1N0M0 | No |
| #7 | Male | 51 | NSCLC | / | T1N0M0 | No |

NSCLC, non-small cell lung cancer; ICS, inhaled corticosteroids; LABA: long-acting beta_2_-agonist.
